# (1→3)-β-d-Glucan: A Biomarker for Microbial Translocation in Individuals with Acute or Early HIV Infection?

**DOI:** 10.3389/fimmu.2016.00404

**Published:** 2016-10-03

**Authors:** Martin Hoenigl, Josué Pérez-Santiago, Masato Nakazawa, Michelli Faria de Oliveira, Yonglong Zhang, Malcolm A. Finkelman, Scott Letendre, Davey Smith, Sara Gianella

**Affiliations:** ^1^Department of Medicine, Division of Infectious Diseases, University of California San Diego, San Diego, CA, USA; ^2^Section of Infectious Diseases and Tropical Medicine, Department of Internal Medicine, Medical University of Graz, Graz, Austria; ^3^Department of Internal Medicine, Division of Pulmonology, Medical University of Graz, Graz, Austria; ^4^Department of Medicine, AntiViral Research Center, University of California San Diego, San Diego, CA, USA; ^5^Clinical Development, Associates of Cape Cod, Inc., Falmouth, MA, USA; ^6^Department of Neurosciences, HIV Neurobehavioral Research Center, University of California San Diego, San Diego, CA, USA

**Keywords:** beta-d-glucan, HIV, microbial translocation, *Lactobacillales*, microbiome, acute HIV infection

## Abstract

**Background:**

The extent of gut microbial translocation, which plays roles in HIV disease progression and non-AIDS comorbidities, appears to vary with the composition of the gut microbiome, particularly the presence of *Lactobacillales*, which reduce mucosal injury. While low proportions of *Lactobacillales* in the distal gut microbiome are a very promising indicator of microbial translocation, measurement is expensive and complicated and not feasible for clinical routine. (1→3)-β-d-Glucan (BDG) is a component of most fungal cell walls and might be a surrogate marker for *Lactobacillales* proportion in the gut and a useful indicator of HIV-associated gut injury. This study evaluated BDG as a biomarker of gut integrity in adults with acute or early HIV infection (AEH).

**Methods:**

Study samples were collected longitudinally during study visits at weeks 0, 12, and 24 in a cohort of 11 HIV-infected men starting antiretroviral therapy during AEH. Blood plasma levels of BDG, soluble cluster of differentiation 14 (sCD14) and lipopolysaccharide (LPS) were measured and then correlated with the proportion of *Lactobacillales* in the distal gut microbiome, as measured by 16s rDNA sequencing by using mixed-effects models with random intercepts.

**Results:**

Mean BDG and sCD14 levels across subjects were associated with *Lactobacillales* after controlling for time effects and within-subjects correlations (*p*-values < 0.05), while LPS levels were not. Specifically, each point increase in mean BDG and sCD14 levels across participants was associated with 0.31 ± 0.14 and 0.03 ± 0.01 percent decrease in mean *Lactobacillales* proportions, respectively.

**Conclusion:**

BDG and sCD14 may be indicators of low *Lactobacillales* in the gut in adults with acute or early HIV infection, and serve as biomarkers of gut integrity and microbial translocation in HIV infection. Larger studies are needed to confirm our findings.

## Introduction

CD4^+^ T cells trigger many elements of the immune response, including regulation of CD8^+^ T cell activation (1). Early HIV infection is characterized by a dramatic depletion of CD4^+^ T cells and impaired polarization of Th17 cells in the gastrointestinal tract and a massive expansion of activated CD8^+^ T cells causing CD8^+^ T cell-mediated enteropathy (2–4), which is also characterized by microbial overgrowth and translocation of microbial products, including bacteria, fungi, and viruses, from the gut into the systemic circulation (5). *Lactobacillales* influence gut mucosal immunity by increasing the suppressive function of CD4^+^ regulatory T cells of colon lamina propria, which can alleviate HIV-associated colitis (1). High proportions of *Lactobacillales* in the distal gut microbiome appear to reduce mucosal injury, lower inflammatory responses, reduce barrier disruption (6, 7), and may result in less microbial translocation during HIV infection (4, 8, 9). Low proportions of *Lactobacillales* may, therefore, serve as an indicator of microbial translocation, which likely plays a role in HIV disease progression and non-AIDS comorbidities, even when antiretroviral treatment is initiated early in the course of infection (10–12). However, determination of gut *Lactobacillales* proportion is expensive and complicated. Surrogate markers for *Lactobacillales* proportion in the gut microbiome are, therefore, needed for the clinical routine.

The polysaccharide (1→3)-β-d-glucan (BDG) is a cell wall component of most fungal species, and is used as a serum biomarker for early diagnosis of invasive fungal infections (13–15). BDG is not highly specific for fungal infections, however, and among individuals with HIV infection but without invasive fungal infection, elevated levels of BDG in serum correlate with HIV-associated immunosuppression, inflammation, and cardiopulmonary comorbidity (16, 17). Consistent with these links, a recent report indicates that BDG may be a promising biomarker for neurocognitive impairment in virally suppressed HIV-infected adults (18). The pathogenic mechanism behind this finding remains unclear.

We hypothesized that in the absence of invasive fungal infection, BDG may be a biomarker of gut mucosal barrier disruption (19, 20) and microbial translocation (21), potentially contributing to HIV-associated morbidity (22). The objective of this pilot study was to evaluate BDG as a marker of gut permeability by correlating blood BDG with proportions of *Lactobacillales* in the distal gut microbiome of individuals diagnosed with acute and early HIV infection.

## Materials and Methods

In this longitudinal observational analysis, we retrospectively measured levels of BDG in blood plasma samples in a cohort of adults with acute and early HIV infection, and compared BDG levels and established biomarkers of microbial translocation with 16s rDNA sequencing of the gut microbiome.

All 13 individuals participated in the San Diego Primary HIV Infection Research Consortium (SD PIRC), which is composed of individuals diagnosed with acute or early HIV-infection (acute: HIV nucleic acid amplification testing+/antibody- consistent with infection <30 days; early: HIV antibody+/detuned HIV Ab consistent with infection <70 days) followed longitudinally (23–25). The UCSD Human Research Protections Program approved the study protocol, consent document, and all study procedures. All participants provided voluntary, written informed consent. All participants started ART within 2 weeks of study enrollment with a combination of tenofovir, emtricitabine, and ritonavir-boosted atazanavir, with or without maraviroc according to a randomization schedule at entry (baseline). Paired anal swabs [anal swabs have been shown to produce highly reproducible microbiota profiles resembling the human gut microbiota (26)] and blood samples were collected sequentially during study visits at baseline and at weeks 12 (±2 weeks) and 24 (±6 weeks) between August 2010 and September 2011 at the University of California, San Diego and stored at −80°C on the day of collection (4).

(1→3)-β-d-glucan was measured by the Fungitell assay in June 2015 at the Associates of Cape Cod, Inc., research laboratories (Associates of Cape Cod, Inc, East Falmouth, MA, USA). Both comparator biomarkers were measured in 2012 and have been – in part – published before (4). Soluble cluster of differentiation 14 (sCD14) was measured by immunoassay (Quantikine, R&D Systems, Minneapolis, MN, USA). Lipopolysaccharide (LPS) was measured by the Limulus Amebocyte Lysate QCL-1000 assay.

DNA extractions from anal swabs were performed in 2012 using the QIAamp Stool DNA kit (Qiagen) (4). Amplification of bacterial DNA and pyrosequencing amplification of the V6 hypervariable region of the 16 S rDNA gene was carried out in a 50 μl reaction using the highly purified Amplitaq Gold Low DNA polymerase (Applied Biosystems, Foster City, CA, USA) (4). For classification of bacteria, we kept bacterial sequences with at least 90 continuous base pairs with a quality score of at least 20 for further analyses as described before (4).

Statistical analyses were performed using SPSS 22 (SPSS Inc., Chicago, IL, USA). As study samples were collected longitudinally (i.e., repeated measures) we applied mixed-effects models with random intercepts to test whether *Lactobacillales* proportions were associated with between-subjects (Bw) as well as within-subjects (Wi) BDG levels after adjustment for repeated measures. The same analysis was repeated for other biomarkers (sCD14, LPS, and CD4^+^ T cell counts). We used mixed-effects models to analyze our repeated-measures data because its maximum-likelihood estimation of the missing values allows for retaining the participants with missing values. In addition, the mixed-effects model approach increases power to detect effects and allows for a more accurate estimate of correlations. In addition, we also calculated cross-sectional correlations between BDG, *Lactobacillales* and other biomarkers using Pearson correlation coefficient for weeks 12 and 24 and Spearman correlation coefficient for baseline due to skewed distributions at week 0 (i.e., secondary analysis).

## Results

Thirteen men diagnosed with acute (8/13) or early (5/13) HIV infection enrolled. Median age was 29 years (range 21–55 years). About a third (4/13) reported Hispanic ethnicity, the rest reporting White (7/13) or Asian race (2/13). Eleven of the 13 men completed their 12- and 24-week visits. All 11 initiated ART between baseline and week 12, 5/11 (45%) achieving viral suppression (i.e. <50 copies/mL) by week 12, and 8/11 (73%) by week 24. Participants who were not virally suppressed (i.e., HIV RNA >50 copies/mL) at week 12 (6/11) showed a trend for higher BDG levels in plasma (median 52 pg/mL, range 12–122 pg/mL) when compared to those who were virally suppressed (median 9 pg/mL, range 3–46 pg/mL; *p* = 0.052, Mann–Whitney *U* test), while there was no difference at week 24. In addition, no difference in the levels of BDG was observed between participants with and without maraviroc (*n* = 7; median 39, range 13–96 vs. *n* = 6; median 39, range 20–89; *p* > 0.20 at all timepoints). At baseline, BDG levels did not differ between participants with early HIV infection (median 63 pg/mL, range 13–89 pg/mL) and those with acute HIV infection (median 37 pg/mL, range 20–96 pg/mL).

**Figure 1:**
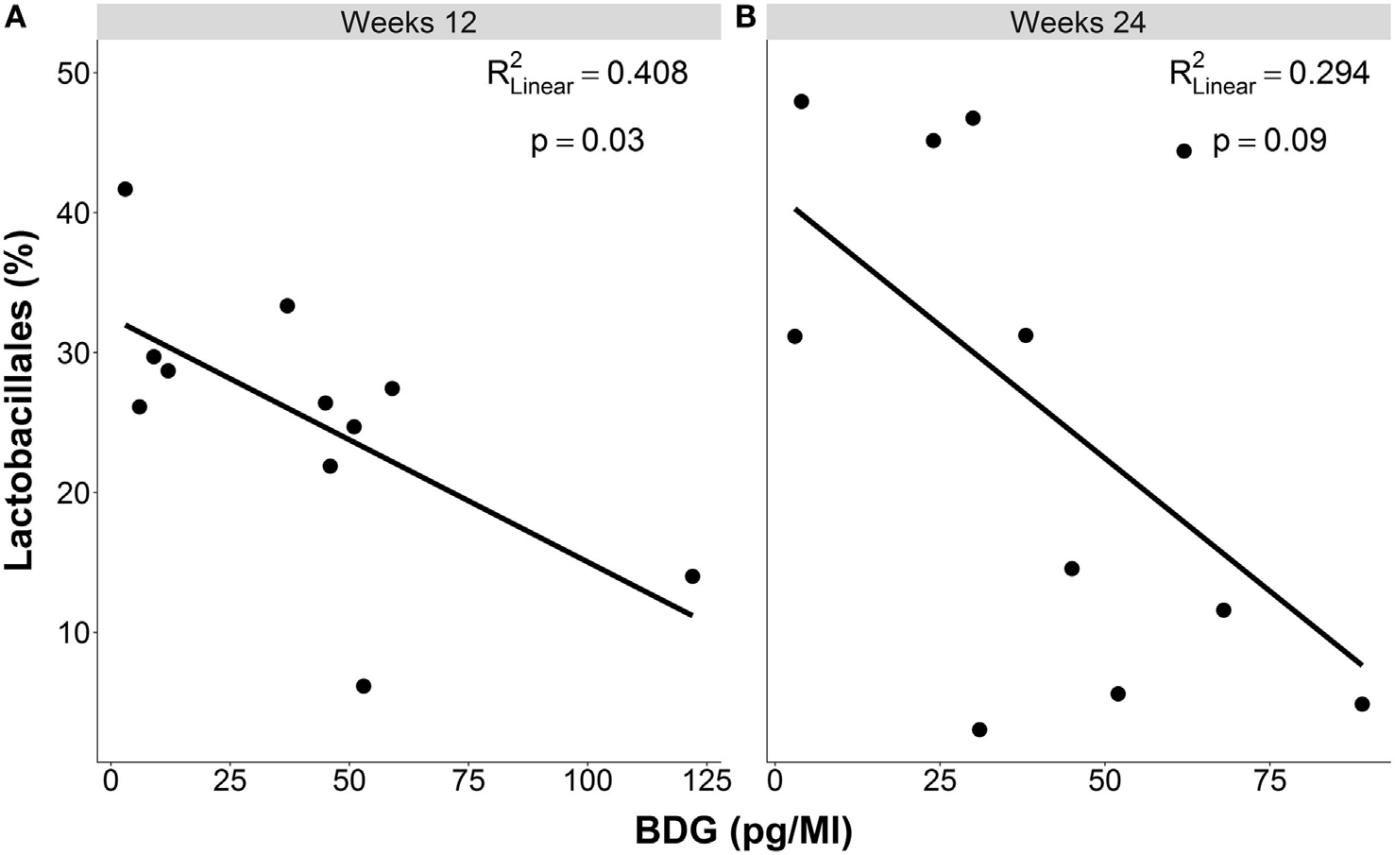
**Cross-sectional associations between levels of beta-d-glucan and proportions of *Lactobacillales* in the distal gut (A) at week 12 (B) at week 24 of follow up in individuals with acute or early HIV infection**.

Levels of clinical and immunological variables as well as the correlation analyses for each time point separately are depicted in Table 1. The median proportion of *Lactobacillales* in the distal gut was 41.5% [range 5.2–52.1%; *n* = 11 (two individuals who received systemic antibacterial therapy were excluded)] at baseline, 26.4% (range 6.2–41.7%; *n* = 11) at week 12, and 22.8% (range 3.0–47.9%) at week 24. While in cross-sectional analysis, there was no correlation between levels of BDG and *Lactobacillales* at baseline, there was a negative correlation at week 12 and a trend at week 24. Scatter plots showing correlations of blood BDG levels and proportions of *Lactobacillales* at weeks 12 and 24 are displayed in Figure 1.

**Table 1:** **Levels of plasma beta d-glucan (BDG) and cross-sectional correlations with other biomarkers, viral loads, and *Lactobacillales* proportions of distal gut bacterial flora (median and range are displayed) at baseline, week 12, and week 24**.

Biomarkers	Baseline (*n* = 13)			Week 12 (*n* = 11)			Week 24 (*n* = 11)		
	Results [median (range)]	Spearman correlation with BDG	*p*-Value	Results [median (range)]	Pearson correlation with BDG	*p*-value	Results [median (range)]	Pearson correlation with BDG	*p*-value
BDG (pg/mL)	39 (13–96)	–	–	45 (3–122)	–		35 (3–89)		
LPS (pg/mL)	0.358 (0.278–0.409)	0.275	n.s.	0.306 (0.278–0.393)	0.263	n.s.	0.311 (0.183–0.411)	−0.241	n.s.
sCD14 (ng/mL)	1,084 (706–2,683)	0.363	n.s	1,290 (728–1,844)	0.352	n.s.	981 (858–1,493)	−0.192	n.s.
CD4^+^ cell count (cells/μL)	520 (91–971)	−0.126	n.s.	822 (245–1,408)	−0.363	n.s.	793 (262–1,150)	−0.456	n.s.
CD8+ cell count (cells/μL)	856 (558–5,216)	−0.357	n.s.	813 (436–2,196)	0.044	n.s.	774 (588–1,906)	−0.009	n.s.
CD4/CD8 ratio	0.576 (0.101–1.279)	−0.055	n.s.	1.077 (0.267–1.372)	−0.475	n.s.	0.888 (0.382–1.070)	−0.493	n.s.
Viral loads (log10 RNA)	5.25 (3.10–7.00)	0.055	n.s.	1.914 (1.623–4.153)	0.147	n.s.	1.681 (1.681–2.702)	0.113	n.s.
*Lactobacillales* (%)	41.49 (5.15-52.12; *n* = 11)	−0.109	n.s.	26.40 (6.16–41.68)	−0.639	0.034	22.85 (3.04–47.94)	−0.542	0.085

**Figure 2:**
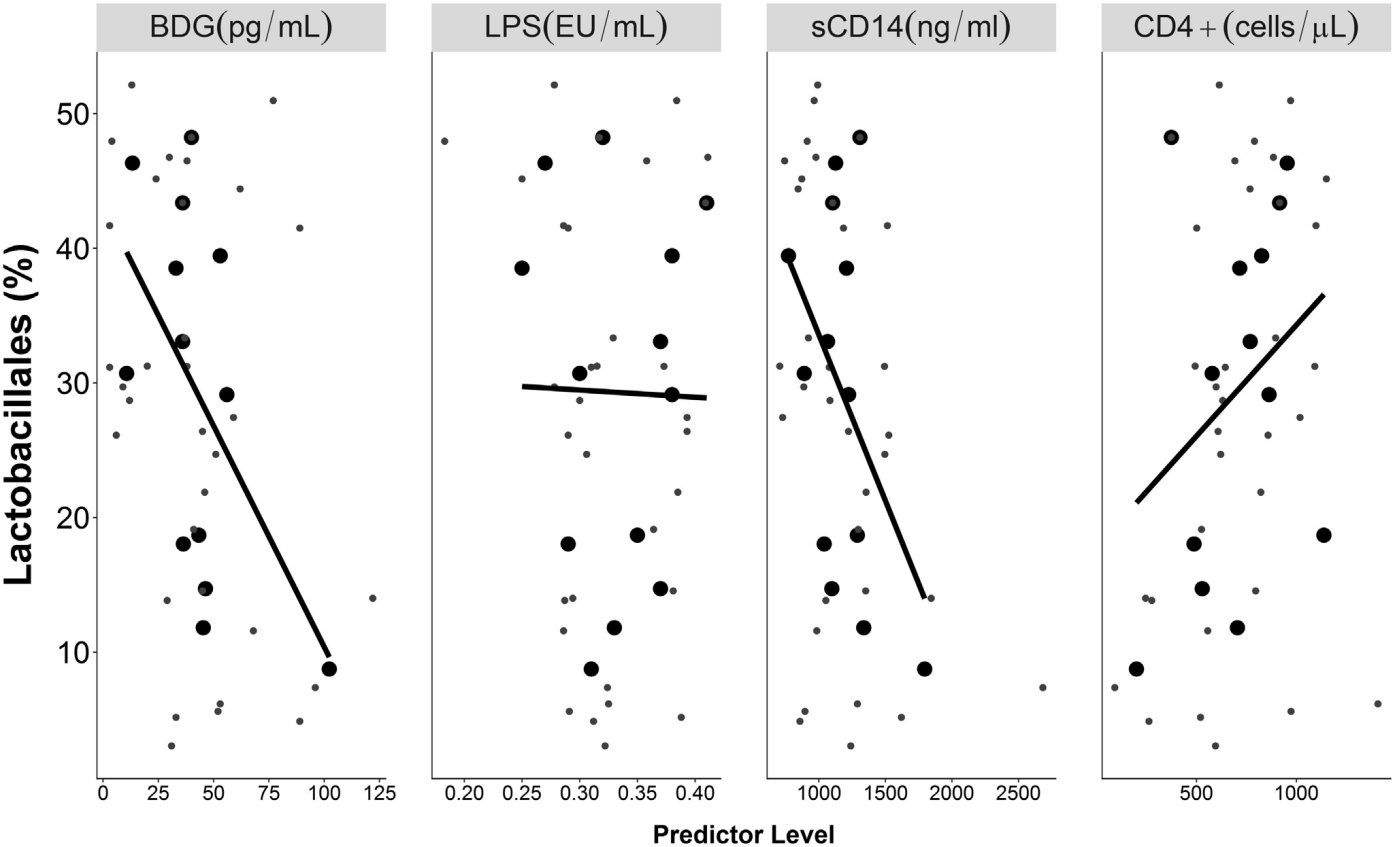
**Longitudinal associations of BDG, LPS, and sCD14 with proportions of *Lactobacillales* proportion in the distal gut**. Big dots indicate mean levels for each subject, and small dots indicate individual observations. Black lines indicate linear regression lines fitted to the mean values, while gray lines indicate non-linear smooth lines.

In the mixed-effects model, higher proportions of *Lactobacillales* were associated with lower Bw BDG (*p* = 0.023, Table 2) and lower Bw sCD14 (*p* = 0.035). Specifically, each point increase in mean BDG and sCD14 levels across participants were associated with 0.31 ± 0.14 and 0.03 ± 0.01 percent decrease in mean *Lactobacillales* proportions, respectively. These two predictors (BDG and sCD 14) were highly correlated (*r* = 0.69, *p* = 0.01). *Lactobacillales* was not associated with Bw LPS (*p* > 0.2) nor with Bw CD4+ cell count (*p* = 0.095). None of the time or Wi effects showed significant associations (*p*-values > 0.2). Scatter plots of mean Wi BDG, LPS, and sCD14 levels predicting *Lactobacillales* proportion in the distal gut are displayed in Figure 2. The big dots are means across longitudinal observations, representing more stable estimates of predictor levels and proportions of *Lactobacillales*. The regression lines fit to those means and, therefore, represent stable estimates of correlations. The small dots indicate individual observations.

**Table 2:** **Summary of mixed-effects models for BDG and other biomarkers predicting *Lactobacillales* proportions**.

	BDG		LPS		sCD14		CD4^+^ cell count	
	β	SMD	β	SMD	β	SMD	β	SMD
(Intercept)	30.47 (4.05)***	3.5	31.04 (4.57)***	3.3	31.91 (4.11)***	3.7	31.12 (4.48)***	3.3
Time	−0.19 (0.23)	0.4	−0.26 (0.25)	0.5	−0.29 (0.23)	0.6	−0.22 (0.27)	0.4
Bw	−0.31 (0.14)*	1.4	−29.03 (79.42)	0.2	−0.03 (0.01)*	1.3	0.02 (0.01)^†^	1.0
Wi	0.05 (0.13)	0.2	−5.61 (86.71)	0.0	−0.01 (0.01)	0.5	0.00 (0.02)	0.0

Bw, between-subjects effect; Wi, within-subjects effect, SMD, standardized mean difference effect-size measure (>0.8 = large effect). Bw and Wi are centered at the grand mean and subject means, respectively. The value inside the parentheses indicates the SE for β. ^†^*p* < 0.10, *p < 0.05, ***p < 0.001 based on likelihood-ratio tests.

## Discussion

This is the first study to evaluate blood levels of the fungal polysaccharide BDG in individuals with acute or early HIV infection. We found that higher levels of blood BDG correlated with higher levels of sCD14 and lower proportions of gut *Lactobacillales*. High proportions of *Lactobacillales* in the distal gut microbiome have been shown to reduce barrier disruption (6, 7), and result in less microbial translocation during HIV infection (4, 8, 9), even when administered as a probiotic treatment (27). Correlation of BDG with high levels of sCD14 and low proportions of *Lactobacillales* may, therefore, suggest that BDG may be a biomarker of gut integrity and microbial translocation in individuals with acute or early HIV infection.

Our findings may have important clinical implications, as microbial translocation may be a major contributing factor to HIV-associated morbidity (22, 28). Two previous studies found that blood BDG levels correlated directly with non-AIDS comorbidities among individuals with chronic HIV infection. Morris and colleagues found that high blood BDG was associated with HIV-associated immunosuppression, inflammation (plasma interleukin-8 and tumor necrosis factor-α), and cardiopulmonary comorbidity in 132 HIV-infected outpatients, the majority of whom had measurable HIV RNA in their blood (17). Our study team recently reported that higher blood BDG levels correlated with worse neurocognitive performance in 19 virologically suppressed HIV-infected adults (18). While both studies explained their findings with the hypothesis that elevated plasma BDG may primarily reflect translocation of products from endogenous fungal flora from the gastrointestinal tract into systemic circulation, this is the first study that actually evaluated BDG as a biomarker of the composition of the gut microbiome, namely *Lactobacillales* (4, 6–8, 22, 29, 30).

In this study, the mean levels of BDG and sCD14 across subjects correlated strongly and were significantly associated with *Lactobacillales* after controlling for time effects and within-subjects correlations, while no significant correlations were found between LPS and *Lactobacillales*. The lack of associations of LPS with other biomarkers of microbial translocation may be explained by the fact that currently available LPS assays rely on limulus amebocyte lysate (LAL) reagents, which are not specific for endotoxin and may be subject to cross-reactivity (31, 32).

Our results indicate that BDG correlates with sCD14 and may be an indicator of low *Lactobacillales* in the gut and increased microbial translocation in adults with acute or early HIV infection. This theory is supported by a number of studies reporting positive BDG levels during hemodialysis (33, 34), most likely explained by transient reduced blood flow within the splanchnic region (35), potentially resulting in ischemia and a transient barrier damage in the gut. By contrast, another recent study did not find elevated serum BDG levels in hematological malignancy patients suffering for mild to moderate mucositis (36).

We also observed a trend toward higher BDG levels in those who were not virologically suppressed at week 12. This finding is in line with results from Morris and colleagues who found that high BDG levels were associated with high viral loads and also low CD4^+^ cell counts among chronically infected individuals (17). While elevated blood BDG levels may be associated with microbial translocation in all HIV-infected individuals (i.e., independent of CD4^+^ cell counts), interpretation of elevated blood BDG levels in individuals with CD4^+^ cell counts below 200–300 cells/μL may be more complicated. While it seems intuitive that deteriorating CD4^+^ counts are associated with worse mucosal barrier function (21), other reasons for elevated BDG may include potential colonization or subclinical infection with *Candida* spp. or *Pneumocystis* (15) that may occur more frequently in individuals with lower CD4 counts.

Major limitations of this small study include its small sample size, its cross-sectional, single-site design, and its focus on adults with acute or early HIV infection. While the mechanistic hypothesis of the correlation between *Lactobacillales* proportion in the gut and microbial translocation has been evaluated in a number of studies, very few studies to date have actually reported that the proportion of *Lactobacillales* may be an indicator of microbial translocation, and the most important study showing this correlation used in part the same samples that were used in this study. Also BDG has been primarily used as a biomarker for fungal infection and BDG cut-off levels for microbial translocation have yet not been defined. However, our study cohort of individuals with AEH presented with markedly higher BDG levels when compared to previously published levels from healthy individuals undergoing elective plastic surgery procedures (37). In addition, cross-sectional analyses of this longitudinal data were limited by particularly small sample sizes. Another explanation for the absence of correlation between BDG levels and *Lactobacillales* in cross-sectional analysis at baseline may be the fact that *Lactobacillales* proportions were markedly lower at week 12 and week 24 of follow-up when compared to baseline (median 22–26% vs. median 41%). The clear negative correlations between BDG and *Lactobacillales* and positive correlations with sCD14 levels in the mixed-effects model suggest nevertheless that BDG may be an indicator of gut mucosal barrier interruption and microbial translocation. To further examine the role of BDG as a potential biomarker for translocation of gut luminal contents, more comprehensive studies will be necessary. BDG levels in the intestinal luminal contents are also likely to be highly variable on an individual basis, and a standardized oral BDG challenge approach may be more suitable for assessing gut integrity.

In conclusion, high BDG levels may be a useful indicator of low *Lactobacillales* in the gut and microbial translocation in individuals with acute and early HIV infection. These findings indicate that BDG may be a promising marker for gut integrity. Larger studies are needed to confirm our findings.

## Author Contributions

MH had the study idea. JP-S, MO, DS, and SG provided the samples for the study. All authors (MH, JP-S, MO, YZ, MF, SL, DS, and SG) contributed to the study design. YZ and MF tested the samples. MH, JS, and SG conducted the data analysis and interpretation. MH, JS, and SG prepared the manuscript. MO, YZ, MF, SL, and DS revised it critical for intellectual contact. All authors have given final approval of the version to be published.

## Conflict of Interest Statement

MH served on the speakers’ bureau of Merck. YZ and MF are employees of Associates of Cape Cod. DS received ViiV Healthcare (Pfizer joint venture) funding. The remaining authors declare that the research was conducted in the absence of any commercial or financial relationships that could be construed as a potential conflict of interest.

## References

[1] Hacini-Rachinel F Nancey S Boschetti G Sardi F Doucet-Ladeveze R Durand PY CD4+ T cells and *Lactobacillus casei* control relapsing colitis mediated by CD8+ T cells. J Immunol (2009) 183:5477–86. 10.4049/jimmunol.0804267 19843933

[2] Arnoczy GS Ferrari G Goonetilleke N Corrah T Li H Kuruc J Massive CD8 T cell response to primary HIV infection in the setting of severe clinical presentation. AIDS Res Hum Retroviruses (2012) 28:789–92. 10.1089/AID.2011.0145 22011008 PMC3399555

[3] DaFonseca S Niessl J Pouvreau S Wacleche VS Gosselin A Cleret-Buhot A Impaired Th17 polarization of phenotypically naive CD4(+) T-cells during chronic HIV-1 infection and potential restoration with early ART. Retrovirology (2015) 12:38. 10.1186/s12977-015-0164-6 25924895 PMC4438463

[4] Perez-Santiago J Gianella S Massanella M Spina CA Karris MY Var SR Gut Lactobacillales are associated with higher CD4 and less microbial translocation during HIV infection. AIDS (2013) 27:1921–31. 10.1097/QAD.0b013e3283611816 24180001 PMC3816380

[5] Klatt NR Funderburg NT Brenchley JM. Microbial translocation, immune activation, and HIV disease. Trends Microbiol (2013) 21:6–13. 10.1016/j.tim.2012.09.001 23062765 PMC3534808

[6] Llopis M Antolin M Guarner F Salas A Malagelada JR. Mucosal colonisation with *Lactobacillus casei* mitigates barrier injury induced by exposure to trinitronbenzene sulphonic acid. Gut (2005) 54:955–9. 10.1136/gut.2004.056101 15951541 PMC1774610

[7] Papoff P Ceccarelli G d’Ettorre G Cerasaro C Caresta E Midulla F Gut microbial translocation in critically ill children and effects of supplementation with pre- and pro biotics. Int J Microbiol (2012) 2012:151393. 10.1155/2012/151393 22934115 PMC3426218

[8] Jenabian MA El-Far M Vyboh K Kema I Costiniuk CT Thomas R Immunosuppressive tryptophan catabolism and gut mucosal dysfunction following early HIV infection. J Infect Dis (2015) 212:355–66. 10.1093/infdis/jiv037 25616404

[9] Dagenais-Lussier X Aounallah M Mehraj V El-Far M Tremblay C Sekaly RP Kynurenine reduces memory CD4 T-cell survival by interfering with interleukin-2 signaling early during HIV-1 infection. J Virol (2016) 90:7967–79. 10.1128/JVI.00994-16 27356894 PMC4988137

[10] Heaton RK Grant I Butters N White DA Kirson D Atkinson JH The HNRC 500 – neuropsychology of HIV infection at different disease stages. HIV Neurobehavioral Research Center. J Int Neuropsychol Soc (1995) 1:231–51. 10.1017/S1355617700000230 9375218

[11] Hunt PW. HIV and inflammation: mechanisms and consequences. Curr HIV/AIDS Rep (2012) 9:139–47. 10.1007/s11904-012-0118-8 22528766

[12] Kelesidis T Kendall MA Yang OO Hodis HN Currier JS. Biomarkers of microbial translocation and macrophage activation: association with progression of subclinical atherosclerosis in HIV-1 infection. J Infect Dis (2012) 206:1558–67. 10.1093/infdis/jis545 23066162 PMC3475633

[13] Reischies FM Prattes J Pruller F Eigl S List A Wolfler A Prognostic potential of 1,3-beta-d-glucan levels in bronchoalveolar lavage fluid samples. J Infect (2016) 72:29–35. 10.1016/j.jinf.2015.09.016 26416472

[14] Reischies FM Prattes J Woelfler A Eigl S Hoenigl M. Diagnostic performance of 1,3-beta-d-glucan serum screening in patients receiving hematopoietic stem cell transplantation. Transpl Infect Dis (2016) 18:466–70. 10.1111/tid.12527 26992092

[15] Prattes J Hoenigl M Rabensteiner J Raggam RB Prueller F Zollner-Schwetz I Serum 1,3-beta-d-glucan for antifungal treatment stratification at the intensive care unit and the influence of surgery. Mycoses (2014) 57:679–86. 10.1111/myc.12221 25040144

[16] Hoenigl M Faria de Oliveira M érez-Santiago JP Zhang Y Woods SP Finkelman M Correlation of (1→3)-β-D-glucan with other inflammation markers in chronically HIV infected persons on suppressive antiretroviral therapy. GMS Infect Dis (2015) 3:Doc03 10.3205/id000018 PMC5132183 27917362

[17] Morris A Hillenbrand M Finkelman M George MP Singh V Kessinger C Serum (1 – >3)-beta-D-glucan levels in HIV-infected individuals are associated with immunosuppression, inflammation, and cardiopulmonary function. J Acquir Immune Defic Syndr (2012) 61:462–8. 10.1097/QAI.0b013e318271799b 22972021 PMC3494803

[18] Hoenigl M Oliveira MF Perez-Santiago J Zhang Y Morris S McCutchan AJ (1 – >3)-beta-D-Glucan levels correlate with neurocognitive functioning in HIV-infected persons on suppressive antiretroviral therapy: a cohort study. Medicine (Baltimore) (2016) 95:e3162 10.1097/MD.0000000000003162 26986173 PMC4839954

[19] Ellis M Al-Ramadi B Finkelman M Hedstrom U Kristensen J Ali-Zadeh H Assessment of the clinical utility of serial beta-D-glucan concentrations in patients with persistent neutropenic fever. J Med Microbiol (2008) 57:287–95. 10.1099/jmm.0.47479-0 18287290

[20] Shahid Z Sanathkumar N Restrepo A Haider S Muzaffar J Grazziutti M Abstr. Elevated Serum Besta-D-Glucan (BDG) as a Marker for Chemotherapy-Induced Mucosal Barrier Injury (MBI) In Adults with Hematologic Malignancies: A Retrospective Analysis. Boston, MA: Infectious Diseases Society of America (IDSA) (2011). ID Week 2011.

[21] Held J Kohlberger I Rappold E Busse Grawitz A Hacker G Comparison of (1->3)-beta-D-glucan, mannan/anti-mannan antibodies, and Cand-Tec *Candida* antigen as serum biomarkers for candidemia. J Clin Microbiol (2013) 51:1158–64. 10.1128/JCM.02473-12 23363830 PMC3666776

[22] Marchetti G Tincati C Silvestri G. Microbial translocation in the pathogenesis of HIV infection and AIDS. Clin Microbiol Rev (2013) 26:2–18. 10.1128/CMR.00050-12 23297256 PMC3553668

[23] Hoenigl M Green N Camacho M Gianella S Mehta SR Smith DM Signs or symptoms of acute HIV infection in a cohort undergoing community-based screening. Emerg Infect Dis (2016) 22:532–4. 10.3201/eid2203.151607 26890854 PMC4766914

[24] Hoenigl M Anderson CM Green N Mehta SR Smith DM Little SJ. Repeat HIV-testing is associated with an increase in behavioral risk among men who have sex with men: a cohort study. BMC Med (2015) 13:218. 10.1186/s12916-015-0458-5 26444673 PMC4596465

[25] Hoenigl M Weibel N Mehta SR Anderson CM Jenks J Green N Development and validation of the San Diego Early Test Score to predict acute and early HIV infection risk in men who have sex with men. Clin Infect Dis (2015) 61:468–75. 10.1093/cid/civ335 25904374 PMC4542926

[26] Budding AE Grasman ME Eck A Bogaards JA Vandenbroucke-Grauls CM van Bodegraven AA. Rectal swabs for analysis of the intestinal microbiota. PLoS One (2014) 9:e101344. 10.1371/journal.pone.0101344 25020051 PMC4096398

[27] Ortiz AM Klase ZA DiNapoli SR Vujkovic-Cvijin I Carmack K Perkins MR IL-21 and probiotic therapy improve Th17 frequencies, microbial translocation, and microbiome in ARV-treated, SIV-infected macaques. Mucosal Immunol (2016) 9:458–67. 10.1038/mi.2015.75 26286233 PMC4760912

[28] Mehraj V Jenabian MA Ponte R Lebouche B Costiniuk C Thomas R The plasma levels of soluble ST2 as a marker of gut mucosal damage in early HIV infection. AIDS (2016) 30:1617–27. 10.1097/QAD.0000000000001105 27045377 PMC4900419

[29] Klatt NR Chomont N Douek DC Deeks SG. Immune activation and HIV persistence: implications for curative approaches to HIV infection. Immunol Rev (2013) 254:326–42. 10.1111/imr.12065 23772629 PMC3694608

[30] Vyboh K Jenabian MA Mehraj V Routy JP. HIV and the gut microbiota, partners in crime: breaking the vicious cycle to unearth new therapeutic targets. J Immunol Res (2015) 2015:614127. 10.1155/2015/614127 25759844 PMC4352503

[31] Kiers D Gerretsen J Janssen E John A Groeneveld R van der Hoeven JG Short-term hyperoxia does not exert immunologic effects during experimental murine and human endotoxemia. Sci Rep (2015) 5:17441. 10.1038/srep17441 26616217 PMC4663498

[32] Maitra U Chang S Singh N Li L. Molecular mechanism underlying the suppression of lipid oxidation during endotoxemia. Mol Immunol (2009) 47:420–5. 10.1016/j.molimm.2009.08.023 19773084 PMC2834173

[33] Koo S Bryar JM Page JH Baden LR Marty FM Diagnostic performance of the (1 – >3)-beta-D-glucan assay for invasive fungal disease. Clin Infect Dis (2009) 49:1650–9. 10.1086/647942 19863452

[34] Theel ES Doern CD beta-D-glucan testing is important for diagnosis of invasive fungal infections. J Clin Microbiol (2013) 51:3478–83. 10.1128/JCM.01737-13 23850953 PMC3889722

[35] Jakob SM Ruokonen E Vuolteenaho O Lampainen E Takala J. Splanchnic perfusion during hemodialysis: evidence for marginal tissue perfusion. Crit Care Med (2001) 29:1393–8. 10.1097/00003246-200107000-00015 11445693

[36] Prattes J Raggam RB Vanstraelen K Rabensteiner J Hoegenauer C Krause R Chemotherapy-induced intestinal mucosal barrier damage: a cause of falsely elevated serum 1,3-beta-d-glucan levels? J Clin Microbiol (2016) 54:798–801. 10.1128/JCM.02972-15 26719433 PMC4767967

[37] Pruller F Wagner J Raggam RB Hoenigl M Kessler HH Truschnig-Wilders M Automation of serum (1 – >3)-beta-D-glucan testing allows reliable and rapid discrimination of patients with and without candidemia. Med Mycol (2014) 52:455–61. 10.1093/mmy/myu023 24906361

